# Toward Proof of Concept of a One Health Approach to Disease Prediction and Control

**DOI:** 10.3201/eid1912.130265

**Published:** 2013-12

**Authors:** Peter M. Rabinowitz, Richard Kock, Malika Kachani, Rebekah Kunkel, Jason Thomas, Jeffrey Gilbert, Robert Wallace, Carina Blackmore, David Wong, William Karesh, Barbara Natterson, Raymond Dugas, Carol Rubin, for the Stone Mountain One Health Proof of Concept Working Group

**Affiliations:** Yale University School of Medicine, New Haven, Connecticut, USA (P.M. Rabinowitz); University of London, London, UK (R. Kock);; Western University of Health Sciences, Pomona, California, USA (M. Kachani);; Centers for Disease Control and Prevention, Atlanta, Georgia, USA (R. Kunkel, J. Thomas, C. Rubin);; International Livestock Research Institute, Vientiane, Laos (J. Gilbert);; University of Minnesota, Minneapolis, Minnesota, USA (R. Wallace);; Florida Department of Public Health, Tallahassee, Florida, USA (C. Blackmore);; National Park Service, Washington, DC, USA (D. Wong);; Ecohealth Alliance, New York, New York, USA (W. Karesh);; University of California Los Angeles Medical Center, Los Angeles, California, USA (B. Natterson);; Pan American Health Organization/Panaftosa, Santiago, Chile (R. Dugas); 1 Current affiliation: University of Washington, Seattle, Washington, USA.

**Keywords:** One Health, proof of concept, zoonoses, comparative effectiveness research, veterinary medicine, public health, animal health, human health, environmental health, interprofessional relations

## Abstract

A One Health approach considers the role of changing environments with regard to infectious and chronic disease risks affecting humans and nonhuman animals. Recent disease emergence events have lent support to a One Health approach. In 2010, the Stone Mountain Working Group on One Health Proof of Concept assembled and evaluated the evidence regarding proof of concept of the One Health approach to disease prediction and control. Aspects examined included the feasibility of integrating human, animal, and environmental health and whether such integration could improve disease prediction and control efforts. They found evidence to support each of these concepts but also identified the need for greater incorporation of environmental and ecosystem factors into disease assessments and interventions. The findings of the Working Group argue for larger controlled studies to evaluate the comparative effectiveness of the One Health approach.

Recent global disease events have highlighted the increasing effects of zoonotic (transmitted from animals to humans) pathogens on human and animal health (*1*). It has also become evident that changes in the environment, including agricultural intensification, population growth, climate change, and human encroachment into wildlife habitats, are drivers for such zoonotic disease emergence (*2*,*3*) and that environmental contamination with toxic chemicals and other hazards threaten human and animal populations (*4*).

The One Health approach emphasizes the relatedness of human, animal, and environmental health (*5*) and the importance of transdisciplinary efforts (*6*). The One Health approach has been recognized as a major element of disease control and prevention strategies by international agencies, including the Food and Agriculture Organization of the United Nations, the World Organisation for Animal Health, and the World Health Organization (www.who.int/influenza/resources/documents/tripartite_concept_note_hanoi/en/index.html) and by national agencies and professional groups across multiple disciplines in several countries (www.onehealthinitiative.com/supporters.php).

As with any paradigm, there is a need to explore and document the comparative effectiveness of the One Health approach over conventional approaches to disease prevention and control. A recent Institute of Medicine report on microbial threats to global food safety called for “research prototypes for proof of concept validation of One Health principles as applied to food safety in the developing world, and also to public–private partnerships between government and the food industry” (*7*), and a US–Mexico border One Health coalition has recommended a “proof of concept project to validate a One Health approach in preparedness and response to external stakeholders” (www.oneborderonehealth.com).

In the biomedical context, “proof of concept” describes evidence that a particular drug, device, treatment method, or other approach is feasible, effective, and provides added value over existing approaches. Often a proof of concept study is a pilot clinical trial that helps determine whether larger, more definitive studies are indicated. Although this proof of concept model is typical of the investigation of a new drug (*8*), similar methods have been used to assess the proof of concept of novel teaching methods for physicians (*9*), mind–body techniques for stress reduction (*10*), and intersectoral collaboration between health services and fire departments to prevent elderly persons from falling (*11*).

At the Operationalizing One Health meeting, held May 4–6, 2010, in Stone Mountain, Georgia, USA (www.cdc.gov/onehealth/pdf/atlanta/meeting-overview.pdf), a multidisciplinary working group was formed to assess the state of evidence in support of the One Health approach. We describe the review of existing evidence conducted by this working group.

## Methods

### One Health Approach Working Definition

At the onset of discussions, the Working Group identified the need to clarify a working definition of the One Health approach to disease prevention and control that distinguished it from traditional public health or clinical practice and would allow a rigorous examination of evidence for proof of concept. Through a process involving in-person meetings and follow-up circulation and discussion of draft criteria, the Working Group sought consensus about such a working definition. While recognizing that One Health is an evolving approach with historical roots in previous formulations such as One Medicine (*12*), the Working Group noted that recent published definitions of One Health have stressed the critical need for transdisciplinary integration of human, animal, and environmental health (*13*). Therefore, the Working Group specified the following concepts underlying the One Health approach that could be subjected to evidence testing:

1) It is feasible to integrate human, animal, and environmental health efforts to predict and control certain diseases at the human–animal–ecosystem interface.

2) Integrated approaches that consider human, animal, and environmental health components can improve prediction and control of certain diseases.

In forming this working definition, the Working Group did not specify the number or types of professionals involved in a particular effort. We specified only that human, animal, and environmental health aspects of a disease issue be considered and assessed.

### Relevant Studies: Search Methods and Selection Criteria

To identify evidence related to these concepts, in 2012, Working Group members from Yale University and the Centers for Disease Control and Prevention used the OVID search engine to search the MEDLINE database of the National Library of Medicine from 1948 through 2012. We performed similar searches by using Web of Science and Google Scholar. We used the search terms “human,” “animal,” and “environment” and determined the intersections of these 3 terms (“human” and “animal” and “environment”). We then looked for studies that reported on control interventions (“intervention or intervention studies”) and disease prediction (“sentinel” or “prediction”) and finally determined whether there were disease prediction or control studies that were classified as relating to humans, animals, and the environment. The 3 search engines identified 44,598 (MEDLINE), 8,893 (Web of Science), and 1,200,000 (Google Scholar) studies that simultaneously referenced human, animals, and the environment (Figure). When the search terms of “intervention studies” and/or “prediction studies” were added, however, a much smaller number of studies were flagged by the MEDLINE and Web of Science searches. For example, the MEDLINE search flagged 457 integrated prediction studies and 503 integrated intervention studies (Figure). From the results of these final searches, Working Group members manually reviewed all titles and abstracts to identify original English language studies fitting the criteria of any of the following:

**Figure:**
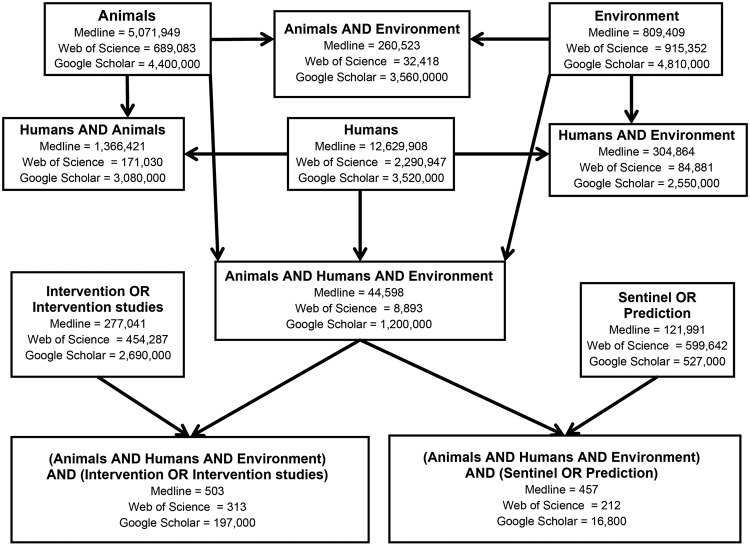
Results of literature search for evidence associated with integration of human, animal, and environmental health.

1. studies demonstrating the feasibility of integrated approaches between human, animal, and environmental health sectors, including collaboration and communication between these 3 sectors;
2. prediction studies that used integrated approaches to predict the risk for disease outbreaks in human and animal populations; and
3. intervention studies that involved human health, animal health, and environmental health/science sectors in the intervention and that assessed outcomes of the intervention in humans AND at least 1 nonhuman animal species AND the environment.

Because of the large volume of citations returned, only a partial review was performed of the citations in the Google Scholar search. Suggestions from Working Group members and review of selected article reference lists led to the identification of additional studies fitting the above criteria. 

For each study included in this review, we noted the method used by the investigators. This information enabled comparison with systems such as the Oxford Levels of Evidence criteria from the Centre for Evidence Based Medicine that consider randomized controlled trials and high-quality systematic reviews to provide a higher level of evidence in support of a treatment or disease control strategy than that provided by case reports or expert opinions (www.cebm.net/index.aspx?o=5653).

Through this process, we identified 5 original studies that primarily provided evidence of feasibility for intersectoral cooperation between human, animal, and environmental health; 6 that provided evidence regarding such collaboration for disease prediction; and 5 that provided evidence regarding disease control (Table).

**Table:** Table. Evidence in support of concepts underlying the One Health approach

One Health Concept	Evidence in support of concept	Study type	Reference
It is feasible to integrate human, animal, and environmental health efforts.	Reports of animal illness facilitated investigation of human cases caused by toxic environmental chemicals.	Case report	*14*
	Animal and human cases of *Cryptococcus gattii* infection can help identify environmental risk for infection.	Case report	*15*
	Collaboration between public health and wildlife health agencies enabled simultaneous testing of bats for rabies and white nose syndrome.	Case report	*16*
	A mathematical model showed proof of concept for an integrated approach to avian influenza control.	Disease model	*17*
	Sheep and cattle deaths helped trace release of weaponized anthrax.	Case report	*18*
Integrated approaches that consider human, animal, and environmental health components can improve prediction of certain diseases.	Cattle poisonings caused by lead exposure in the soil helped detect cases of lead poisoning in humans living nearby.	Case report	*19*
	Household pets served as sentinels for childhood lead poisoning risk.	Case report	*20*
	A household bird provided warning of carbon monoxide poisoning to household members.	Case report	*21*
	A prediction model incorporating bird, mosquito, and climate data was superior to less integrated models for predicting human infection with West Nile virus in Los Angeles, California.	Retrospective case cross-over study	*22*
	Climate based models predicted Rift Valley fever in humans and animals.	Prospective observational study	*23*
	Seasonal temperatures predicted risk for campylobacteriosis in chickens and humans.	Retrospective longitudinal study	*24*
Integrated approaches that consider human, animal, and environmental health components can improve control of certain diseases.	Enhanced mechanized ventilation in a horse stable led to improvements in indoor air quality and in the respiratory health of horses and humans.	Case report	*25*
	Reduced cases of poultry and human campylobacteriosis in Iceland over a multiyear period was attributed to better on-farm biosecurity measures and public education.	Retrospective longitudinal study	*26*
	Rates of human infection with *Schistosoma japonicum* were lower when treatment was given to humans and domestic buffaloes than when treatment was given to humans only.	Controlled intervention study	*27* *,* *28*
	Environmental interventions helped reduce human, animal, and environmental rates of *S. japonicum* infection.	Controlled intervention study	*29*
	The spread of methicillin-resistant *Staphylococcus aureus* infection in a horse hospital was stopped by environmental cleaning and isolation of animals and humans.	Case report	*30*

## Results

### Feasibility Studies

In reviewing evidence for the feasibility of integrating human, animal, and environmental health, the Working Group found studies indicating that linking 2 of these domains (human and animal health) could lead to improved service delivery for humans and animals (Table). Schelling et al. (*31*) reported that joint animal and human vaccination campaigns among nomadic pastoralists in Africa increased the rate of human vaccination; Cleaveland et al. (*32*) found that vaccination of dogs for rabies in Africa reduced the need for postexposure rabies prophylaxis for humans. We found evidence for feasibility of linking human and veterinary services to address not only acute disease threats but also chronic diseases such as obesity. A pilot intervention study found that encouraging exercise for obese persons and their dogs resulted in weight loss and increased physical activity for both (*33*).

Evidence for feasibility of integration across all 3 sectors of human, animal, and environmental health, however, came from other studies, such as an article detailing how reports of animal illness facilitated investigations of human cases of disease caused by chemical hazards in the indoor environment (*14*). Likewise, a recent state-based effort found benefits of working across human, animal, and environmental health sectors for simultaneously tracking risk for rabies and white nose syndrome in bats (*16*), and an effort to track *Campylobacter* infection in poultry found that environmental temperature affected infection risk for broiler flocks and for humans. A mathematical model of avian influenza transmission between wild birds and domestic poultry was used to provide proof of concept for a proposed integrated intervention involving human, animal, and environmental health to interrupt such transmission (*17*). An earlier example of successful collaboration between the human, animal, and environmental health sectors was the investigation of the release of weaponized anthrax from a laboratory in Sverdlovsk, Russia. Analysis of sheep and cattle deaths helped the investigative team trace the airborne spread of the pathogen, because the animals, being more susceptible, died over a wider area than did humans (*18*). This analysis helped establish that the outbreak was caused by airborne release, contrary to the initial attribution of the outbreak to contaminated meat. More recently, human and animal cases of infection with the emerging fungal pathogen *Cryptococcus gattii* showed promise for identifying ecologic niches and areas at high risk for this infection. (*15*).

### Prediction Studies

The Working Group found several case series that showed how an integrated approach could lead to improved prediction of emerging disease threats in the environment (Table). Cattle poisonings resulting from exposure to lead in the soil helped public health professionals detect cases of lead poisoning in humans living nearby (*19*). Similarly, household pets have reportedly served as sentinels for lead poisoning risk among children (*20*). And like the “canary in the coal mine,” domestic birds have been found to provide advance warning of carbon monoxide exposure for children and others in a household (*21*).

In terms of predicting infectious disease outbreaks, an integrated prediction system that used bird, mosquito, and climate data proved to be better than less integrated models for predicting human West Nile Virus infection risk in Los Angeles County, California (*22*). Similarly, climate-based models have helped predict recent outbreaks of Rift Valley fever among humans and animals in Africa (*23*).

### Intervention Studies

The Working Group found only a few studies that reported on integrated disease interventions that considered human, animal, and environmental health. Some of these studies, however, were of high quality (Table).

A study of an intervention to provide mechanized ventilation in a horse barn environment found that the enhanced ventilation led to improvements in the indoor air quality in the barn and to the respiratory health of the horses and humans exposed to the barn environment (*25*). In Iceland, better on-farm biosecurity measures and public education have been credited for reducing poultry and human cases of campylobacteriosis over several years (*26*). And after cases of methicillin-resistant *Staphylococcus aureus* were detected in a veterinary hospital, the outbreak was controlled through interventions to decontaminate the environment, isolate animals, and educate hospital personnel (*30*).

Several studies reported on successful integrated interventions to reduce the rate of zoonotic schistosomiasis (caused by *Schistosoma japonicum*) in China (*17**,**29*). One randomized controlled study found that rates of human infection and environmental contamination were lower in communities in which humans and bovids received treatment than in control communities in which only humans received treatment (*27*). Another controlled study (*29*) found that infection of humans and nonhuman animals and environmental pathogen loads were lower in villages that used environmental interventions (including removing cattle from snail-infested grasslands, building lavatories to improve sanitation, and providing boats with sanitation containers) than in villages that used the usual disease control measures.

As with any type of intervention, not all studies of integrated interventions considering human, animal, and environmental health demonstrated unequivocal success. An intervention study to reduce rates of campylobacteriosis in a village in Peru (*34*) found that rates of *Campylobacter* diarrheal infection of children were higher among intervention households receiving corrals to contain formerly free-ranging chickens than among control households. However, rates of *Campylobacter* colonization were slightly lower among the corralled chickens than among the free-ranging control chickens.

## Discussion

This review of proof of concept for the One Health approach to emerging disease threats provides evidence that transdisciplinary integration among the sectors of human, animal, and environmental health is feasible. Our Working Group also found that simultaneously considering human, animal, and environmental health aspects when attempting to predict and control infectious pathogens and toxic hazards as well as chronic disease risks is beneficial.

Our findings of the feasibility of intersectoral cooperation efforts between human, animal, and environmental health professionals is consistent with recent international policy recommendations for better horizontal integration of such efforts (*35**,**36*). Our finding of evidence in support of a One Health approach to disease prediction based on linking human, animal, and environmental data supports a National Academies report on the need for integrated surveillance to detect emerging global infectious disease threats (*37*). Although animals can clearly serve as sentinels for human health hazards in the environment, cases of disease in humans can also indicate increased risk for animals. A study of the spread of pandemic influenza A(H1N1)2009 virus found that human cases in a country preceded animal outbreaks, indicating “reverse zoonotic” transmission of the virus and that human cases can serve as sentinels for animal risk (*38*). Integrated disease surveillance could therefore help identify infectious disease risk to human and animal populations.

Similarly, our findings of successful intervention studies that involve human, animal, and environmental health in the intervention and outcome assessments raise the possibility that such approaches could be successful in the long run and more sustainable than disease prevention projects with a more human-centric focus (*39*). At the same time, our search of the literature found only a small number of either disease prediction or intervention studies that simultaneously tracked outcomes in humans, animals, and the environment and that seemed to consider the underlying environmental factors affecting humans and animals. Of note, none of the intervention studies (or prediction studies) that the Working Group identified specifically mentioned a One Health approach in their methods. This finding is understandable because the widespread consideration of this approach is relatively recent. However, the fact that the term is not yet in wide use might have limited the ability of our literature search to identify additional studies.

Furthermore, most studies identified were anecdotal case reports. Such studies are considered by systems such as the Centre for Evidence Based Medicine Levels of Evidence Criteria to provide weaker evidence than controlled studies (www.cebm.net/index.aspx?o=5653). We did, however, identify several controlled prediction and intervention studies that showed a benefit of an integrated One Health type of approach over less intersectoral approaches. 

Taken as a whole, however, our review highlights the need for larger, more controlled comparative studies of One Health disease prediction and control strategies. Such studies would provide clearer evidence of benefit over narrower approaches. Specifically, there should be larger implementation studies of surveillance systems integrating human, animal, and environmental data. As such systems are implemented, the effectiveness of such integrated surveillance should be compared with more segregated systems. Likewise, larger controlled intervention trials of One Health approaches for control of several infectious and chronic diseases could more clearly establish the comparative effectiveness of the One Health approach over either single-sector efforts or projects that focus on human and animal health only and do not consider the environmental and ecosystem factors underlying the problem.

As with other models of disease prevention and control, a careful accounting of costs, both short and long term, will be necessary to show the economic benefits of a One Health approach. Costs have been cited as an obstacle to environmental interventions (*40*); however, if addressing environmental causes of disease proves more effective, the costs could be justified. Furthermore, because a One Health intervention could improve health outcomes in humans, animals, and the environment, the cost-savings or benefits of such an integrated approach need to be assessed across multiple sectors. This type of comprehensive evaluation could demonstrate advantages over existing single-sector approaches and is an additional justification for further study of this integrated approach.
